# Understanding how and why the Gene Ontology and its annotations evolve: the GO within UniProt

**DOI:** 10.1186/2047-217X-3-4

**Published:** 2014-03-18

**Authors:** Rachael P Huntley, Tony Sawford, Maria J Martin, Claire O’Donovan

**Affiliations:** 1 European Molecular Biology Laboratory, European Bioinformatics Institute (EMBL-EBI), Wellcome Trust Genome Campus, Hinxton, Cambridge CB10 1SD, UK

**Keywords:** Gene Ontology, Annotation, Function prediction, Misinterpretation

## Abstract

The Gene Ontology Consortium (GOC) is a major bioinformatics project that provides structured controlled vocabularies to classify gene product function and location. GOC members create annotations to gene products using the Gene Ontology (GO) vocabularies, thus providing an extensive, publicly available resource. The GO and its annotations to gene products are now an integral part of functional analysis, and statistical tests using GO data are becoming routine for researchers to include when publishing functional information. While many helpful articles about the GOC are available, there are certain updates to the ontology and annotation sets that sometimes go unobserved. Here we describe some of the ways in which GO can change that should be carefully considered by all users of GO as they may have a significant impact on the resulting gene product annotations, and therefore the functional description of the gene product, or the interpretation of analyses performed on GO datasets. GO annotations for gene products change for many reasons, and while these changes generally improve the accuracy of the representation of the underlying biology, they do not necessarily imply that previous annotations were incorrect. We additionally describe the quality assurance mechanisms we employ to improve the accuracy of annotations, which necessarily changes the composition of the annotation sets we provide. We use the Universal Protein Resource (UniProt) for illustrative purposes of how the GO Consortium, as a whole, manages these changes.

## Introduction

Since its initial publication in 2000 [1] over 5,000 peer-reviewed articles have cited the Gene Ontology (GO)^a^, and each year an increasing number of researchers are using GO to assist them in informing or validating their hypotheses. GO is used both for small- and large-scale applications, for example, providing functional data for a single protein or a GO term enrichment analysis for an entire proteome, as well as by researchers analyzing the changes in GO itself over time and measuring the reliability and comprehensiveness of GO annotations [2].

With this increasing usage, it is imperative that users of GO are well informed as to how GO and its associations to gene products (GO annotations) are created and maintained. Several helpful articles have been published that describe the work of the GO Consortium (GOC) [3-8], but there are certain changes to the ontology and annotation sets that are less widely known among users of the GO.

GO provides almost 40,000 terms across three ontologies describing biological attributes of gene products (October 2013). This includes the 1) molecular functions a gene product performs, 2) the biological processes it is involved in and 3) the cellular components it is located in; each one of these terms may be associated with any number of gene products. These associations are known as 'annotations’ and can be created either manually or automatically. Manual annotations are made by a curator reading full-text primary literature and capturing functional data [4]. Manual annotations provide detailed and specific information and are critical for creating learning sets for automated pipelines. Automatic annotations are made using algorithms that consider gene product properties, such as orthology, domains and sequence similarity [5,9], and they provide a broad coverage of annotation and cover a significantly larger taxonomic range than manual annotations. This difference in coverage is illustrated by the annotation statistics from the database of the UniProt GO Annotation project (UniProt-GOA) that includes GO annotation from all of the GOC members [5]; as of November 2013, GOC provides over 200 million annotations, with around 99% of these being automatically created [10,11].

Many changes are made to both the ontology and annotation sets over time - some of these changes are planned and announced by GOC or its members via mailing lists or release notes [10,12], whereas others are not and reflect ongoing improvements, such as user requests for updates to the ontology [13] or annotations [14], as well as revisions in response to quality assurance checks.

Here we will cover some of the types of changes that can occur on a regular basis and give examples of unplanned changes that have happened in the past. We will also describe the quality assurance mechanisms we have in place, which are available to any group generating GO annotation, and which can be used to improve the accuracy of both manual and automatic annotations, inevitably changing the composition of GO annotation datasets.

## Review

### Changes to ontologies and annotations

One of the major misconceptions about GO is that the ontologies and annotations give a complete coverage of biological knowledge and are therefore stable and unchanging. This is not the case, partly because biological knowledge itself is incomplete and partly because of the large volume of experimental evidence that has yet to be captured by functional annotation. Changes to both the ontology and annotations are frequent; the revisions and additions that are made to the ontology are publicly released by the GOC each day and those to the UniProt GO annotation dataset every week. At its most complete, GO can only ever reflect what is currently known and there are parts of biology that are not represented in GO as well as they could be. In the case of the ontologies, these parts are being identified and progressively improved by collaborations between expert scientists and the GOC to accurately represent specific areas of biology. These changes to the GO also involve a subsequent effort to assign the new terms to gene products, thereby affecting the composition of annotation sets, in terms of both adding and removing annotations. It should be said, however, that the fact that an association between a gene product and a particular GO term may be removed does not necessarily imply the annotation was incorrect. Here we will describe several reasons why GO terms and annotations may change over time.

### Development of the ontologies

The ontologies need to be refined constantly in order to keep up with the latest biological knowledge and to intersect appropriately with other ontologies. The priorities for development are decided based on the particular interests and expertise of GOC members, funding from external bodies to develop a certain area of the ontology, as well as addressing inconsistencies in the GO. It must be noted that any change to the ontology is carefully considered, and discussed with experts if necessary, to avoid introducing inconsistencies or incorrect information. The ontology request tracker [13] is a publicly available tool for users to request and follow updates to the ontology, and terms affected by a current ontology development project are highlighted in the “GO Discussions” section of the term page in the UniProt-developed GO browser QuickGO [15].

The alterations may involve only small-scale changes to update a definition or add parent or child terms, or it may be a more comprehensive project involving experts in the scientific community to assist a larger restructuring of specific parts of the ontologies. Some examples of this large-scale ontology development that have been done recently include the restructuring and supplementation of the GO terms referring to heart [16] and kidney [17] development, apoptosis [Paola Roncaglia *et al*., personal communication] and the cell cycle [Valerie Wood *et al*., personal communication].

**Figure 1:**
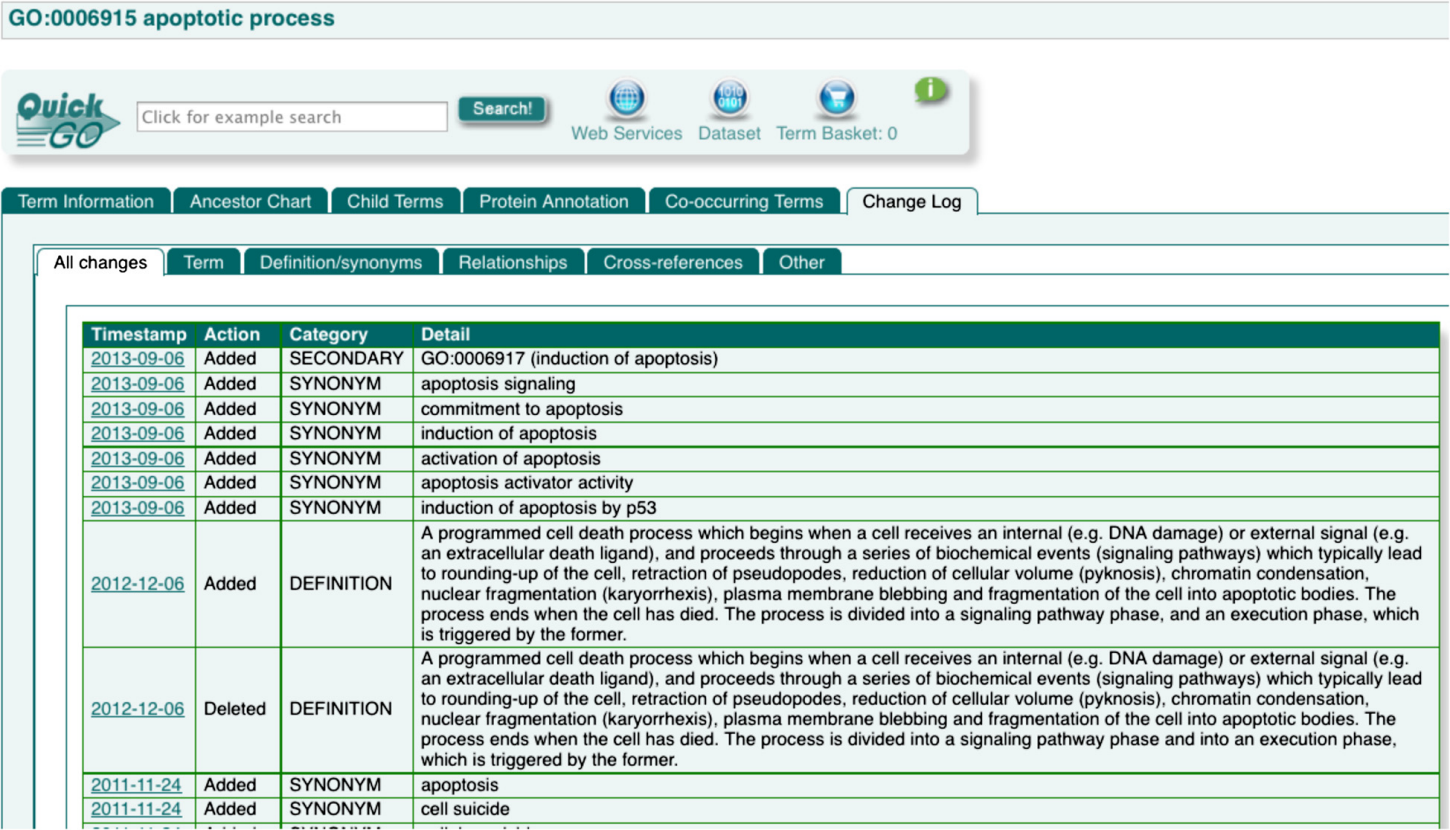
**Changes to the “apoptotic process” term.** The most recent changes to the GO term “apoptotic process” as displayed in QuickGO [20]. In total there have been 54 changes over the lifetime of the term.

If we look at the term 'apoptotic process’ (GO:0006915), we can see how this re-structuring can impact both the ontologies and annotations (Figure 1). During its lifetime, this term has been edited 54 times so far. Most of these are simple changes such as additional synonyms, but there are also refinements to the definition to clarify the scope of the term in response to the experimental knowledge gained over time about this complex process. The restructuring of the apoptosis node in GO resulted in several new, more specific terms and therefore, a re-annotation effort was necessary to reflect the current experimental knowledge in this area. During the re-annotation exercise, annotations were moved to more descriptive terms that were not available at the time of the original annotation; for example 'positive regulation of extrinsic apoptotic signaling pathway via death domain receptors’ (GO:1902043). It is important that users are aware of the regular work the GOC does to improve the annotation of gene products in a certain area of biology because when an annotation is moved to a more granular term, it could be interpreted that the annotation to the less granular term was removed because it was incorrect, when in fact it was an appropriate annotation, but a more specific one could be made instead. The less granular annotation was correct because the GO adheres to the “true-path-rule”, which means that if a gene product is annotated to a given term, it must also be the case that it can be correctly described by all of the ancestors of that term. The introduction of more detailed GO terms, and the subsequent use of these terms for curating gene products, allows the user to identify very specifically the functional role(s) of their proteins of interest.

### Changes to relationships between ontology terms

In addition to updating the ontology terms themselves, changes to relationships between the terms can also have a significant impact on annotations. In November 2011, there was a decrease of ~2,500 manually and automatically assigned annotations to the Biological Process term 'transcription, DNA-dependent’ (GO:0006351; 18% of total GO Consortium inferred annotations: data from UniProt-GOA releases 101 and 100) due to the removal of an inter-ontology link between this term and the Molecular Function term 'sequence-specific DNA binding transcription factor activity’ (GO:0003700). Inter-ontology links are provided when a function is always found to be part of a process, or a process always occurs in a specific subcellular location; these are used to create inferred annotations to Biological Process or Cellular Component terms when a linked Molecular Function or Biological Process term has been annotated, respectively. In the example given, it was determined that gene products involved in sequence-specific DNA binding transcription factor activity are not always directly involved in DNA-dependent transcription and so the link was removed, resulting in the removal of annotations inferred from this ontology link.

### Obsoleting terms from the ontologies

In addition to adding new GO terms and refining existing ones, there is occasionally the need to make obsolete terms that are now considered out-of-scope for GO, for example, those that describe gene products or temporal events. When terms have been identified for obsoletion, quite often a replacement or suggested term is given, for example the Molecular Function term 'apoptosis inhibitor activity’ (GO:0008189) was obsoleted because it was actually describing a Biological Process, and it was therefore replaced by the term 'negative regulation of apoptotic process’ (GO:0043066), meaning any gene products that were associated with the former term are also appropriately described by the latter term. When providing replacement terms, the existing annotations are carefully reviewed to ensure that incorrect annotations will not be introduced. Curators are alerted to any change that may cause problems and are asked to re-annotate to a more appropriate term. Consequently, these refinements can have a significant impact on annotations in terms of numbers, that is, annotations using a term that is now obsolete will be removed and potentially replaced by a more appropriate term, but also an increase in accuracy of the resulting annotations.

### Identifying ontology terms unsuitable for direct annotation

Since 2012, GO has started designating some GO terms as not acceptable for direct annotation (direct annotation here means the use of the GO term as the primary annotation for a gene product). These terms remain in the GO as they are still in scope and are useful for other applications.

There are two such subsets of terms:

1. Those that are inappropriate for both manual and automatic annotation, for example, 'nuclear part’ (GO:0044428) or 'S phase’ (GO:0051320) [18]. The 'Cellular Component-part’ terms are present only for ontology completeness, whereas the cell-cycle phase terms describe a time period rather than a specific process, but remain in the Biological Process ontology as they are used in other parts of an annotation, such as annotation extensions [4], but cannot be used to directly associate to a gene product. For annotations that are associated with a term from this subset, it should always be possible to associate the gene product with an alternative term, for example, gene products associated with 'nuclear part’ (GO:0044428) are more appropriately associated with the 'nucleus’ term (GO:0005634).

2. Those that are inappropriate for manual annotation, but automatic annotation is acceptable (e.g. 'response to stress’ [GO:0006950]) [19]. These terms are considered too general to be useful. In all cases, there should be a more specific term that the gene product should be associated with, for example, a paper describing a gene product’s involvement in a stress response should always detail the type of stress applied, allowing the curator to choose a more specific child term of 'response to stress’ (GO:0006950). Predictions made by automatic annotation methods use these terms as it may not be possible to choose a more specific term that will always be true for all gene products annotated by the prediction method.

Terms in these subsets are identified by a “Restriction” comment in the UniProt-developed GO browser QuickGO [20] alerting curators not to use them.

### Adding annotations using the ontologies

Subsequent to the ontology developments for heart [16] and kidney [17] development, and apoptosis that were described earlier, curators made use of the revised and expanded ontologies to improve the annotation of gene products. In the case of the heart development annotation project, this has so far resulted in almost 30,000 annotations being provided for 4,000 proteins (data from UniProt-GOA release 124). The added value of these new annotations has previously been demonstrated by performing GO term enrichment analyses on annotation sets from before and after the curation project. The analysis of the annotations after the curation effort provided terms with an increased depth and specificity compared with the analysis before the curation effort, thereby improving the interpretation of analyses of large datasets [21,22].

Curators not only provide annotation following ontology development, but also take part in focused annotation of gene products independent of ontology development. As increasing amounts of published experimental evidence has yet to be captured by functional annotation, prioritization of gene products for curation is important to consider. UniProt prioritizes annotation based on the expected benefit to the larger scientific community and has had proven success when curating proteins involved in specific organ development [17,21] or location in a specific organelle [23]. This latter project involved the curation of all known roles and locations of human proteins that are found in the peroxisome. During the course of the curation, 49 new peroxisome-related terms were identified for addition to the ontologies, highlighting that an important role of the curator is to identify and request the creation of terms missing from GO. The project resulted in 1,551 annotations being created for 88 peroxisomal proteins, as well as 296 non-peroxisomal proteins where functional data was available in the same publications as the peroxisomal proteins [23]. This work additionally demonstrated an increased depth and specificity of enriched GO terms in a term enrichment analysis.

### Changes to the reference of annotations

All GO annotations require a reference or authority describing where the evidence for the annotation originated, such as a PubMed identifier or an abstract where a description of how the annotation was made is provided. The latter are termed “GO references*”*[24]. In the process of refining annotation sets, it has very occasionally become necessary to change the reference for a set of annotations in order to better describe the origin of the annotations.

There were two such cases at the beginning of 2013 when it became necessary to change the reference associated with a large set of manually created Cellular Component annotations provided by the Human Protein Atlas and LifeDB projects [25,26]. Previously these annotations were referenced by publications describing the experimental methods used in pilot studies for obtaining the annotations. As such, these publications did not actually contain the experimental data for all of the gene product subcellular localizations that were referenced by them. As this left the annotations open for misinterpretation [27], it was decided that they would be more correctly described using a GO reference. This kind of change is important for users to be aware of since any analysis that makes use of the reference field of an annotation will have registered a large decrease in the number of annotations, and has prompted some researchers to mistakenly assume these annotations were incorrect. When substantial changes such as these are made in the UniProt annotation files, an announcement is always provided with the accompanying file release notes, and we encourage users and researchers to read them to ensure that their subsequent analyses benefit [10].

### Changes in submitted annotation sets

In addition to the planned changes described above, there may be problems with the data supplied by contributing annotation groups that may result in large changes in the number of annotations we import from these external databases. This can be due to a variety of reasons, for example in April 2013 Ensembl Plants suspended GO annotation for some species whilst they introduced taxon constraints into their annotation pipeline. This resulted in the omission of approximately 125,000 automatic annotations from release 119 of the UniProt GO annotation file, and the GO annotations for these species were not available again until release 122 (July 2013), after Ensembl Plants resumed their GO annotation.

Large changes in annotation numbers can also occur when annotating groups update their files that map between their gene product identifiers and UniProtKB accessions. In January 2012, the Zebrafish Model Organism Database updated their identifier mapping file resulting in a decrease of approximately 15,000 manual annotations to zebrafish proteins in release 104 of the UniProt GO annotation file; in November 2011 a similar update to the Rat Genome Database identifier mapping file was responsible for a loss of approximately 20,000 manual annotations to rat proteins (release 100 of the UniProt file).

Given the many changes and updates to the ontologies and annotations described here, no single annotation file can be considered as definitive. It is therefore recommended that if a user wishes to use specific annotation files for analyses, it is first worth checking with the provider of the file to determine if there are any significant changes to the datasets that should be considered.

### Quality assurance

Around 99% of GO annotation in the UniProt-GOA database consist of automatic predictions. This type of annotation is critical for supplying functional information to a wide range of species that do not have experimental data or a dedicated manual annotation focus. There are approximately 31 million proteins spanning 434,561 taxa (October 2013) where the only source of GO annotation is from automatic methods, some examples of which are shown in Table 1. When comparing this with the approximately 264,000 proteins over 2,800 taxa that additionally have manual annotation, it is clear that automatic annotation is a very powerful method of populating large numbers of proteins with annotations in a short amount of time. However we also need to ensure that annotations from these large-scale automatic methods, as well as the manual annotations made by curators, are appropriate and accurate for the species being annotated.

**Table 1:** Examples of taxonomic groups whose only source of annotation is from automatic prediction methods

**Common name**	**Taxon ID**	**No. annotations**
***Viruses***		
Simian immunodeficiency virus	11723	202000
Dengue virus type 1	11053	82000
***Vertebrates***		
West Indian ocean coelacanth	7897	76000
Black flying fox	9402	66000
***Plants***		
Wild banana	214687	82000
Brachypodium distachyon	15368	75000
***Insects***		
Monarch butterfly	13037	36000
Parasitic wasp	7425	35000
***Bacteria***		
Ralstonia solanacearum	305	200000
***Protozoans***		
Paramecium tetraurelia	5888	66000
***Fungi***		
Penicillium stipitatum	441959	35000

This information was obtained by querying the UniProt-GOA database (October 2013).

In addition to the ongoing work to improve existing annotations as described in previous sections, specific taxon-related quality control mechanisms have been developed that can prevent unsuitable annotations from being created in the first place. A description of two of these follows.

### Taxon constraints

GO terms are generally defined to be taxon neutral, but some are applicable only to certain taxa. In 2010, the GOC started applying taxon restrictions to certain GO terms. These restrictions improve the accuracy of annotations as well as identifying errors in the ontologies when applied as an automated check of GO term:taxon combinations. In the initial implementation of the taxon restrictions, approximately 1.6 million erroneous annotations were found and corrected [28]. One example of an improvement to GO, resulting from taxon restrictions, was the refinement of the definitions for the terms concerning microtubule organizing centers (MTOC). In fungi, the MTOC is called the spindle pole body, whilst in mammals it is called the centrosome. In GO we have terms for 'centrosome organization’ (GO:0051297) and for 'spindle pole body organization’ (GO:0051300); only fungal gene products should be annotated to the 'spindle pole body organization’ (GO:0051300) class, therefore the definitions of these terms were clarified so that the meaning is more apparent for curators and users.

The taxon restrictions are publicly available [29,30] and here we explain how to use these effectively. There are currently two types of taxon restrictions; 'only_in_taxon’ or 'never_in_taxon’ and a term can have more than one taxon constraint. It is important to understand that the taxon restrictions are inherited by any child terms of the term they are applied to. For this reason, the taxon restrictions must be used in conjunction with the GO and a taxonomy hierarchy.

**Figure 2:**
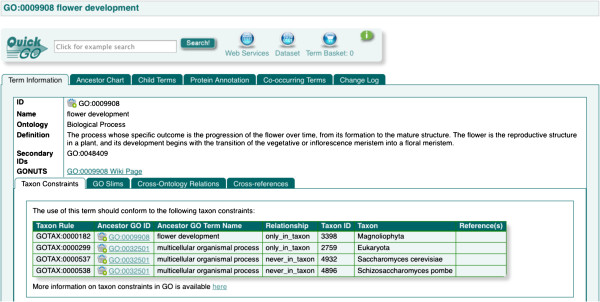
**Taxon restrictions for the term “flower development”.** This term has four taxon restrictions, three of which are inherited from a parent term. These restrictions can prevent GO terms from being used inappropriately for certain taxonomic groups.

For example the term 'flower development’ (GO:0009908) is covered by four taxon restrictions as shown in Figure 2. Only one of these is applied directly to the term itself (flower development can be found *only_in_taxon* Magnoliophyta), the other three restrictions are inherited from the parent term 'multicellular organismal process’ (GO:0032501).

**Figure 3:**
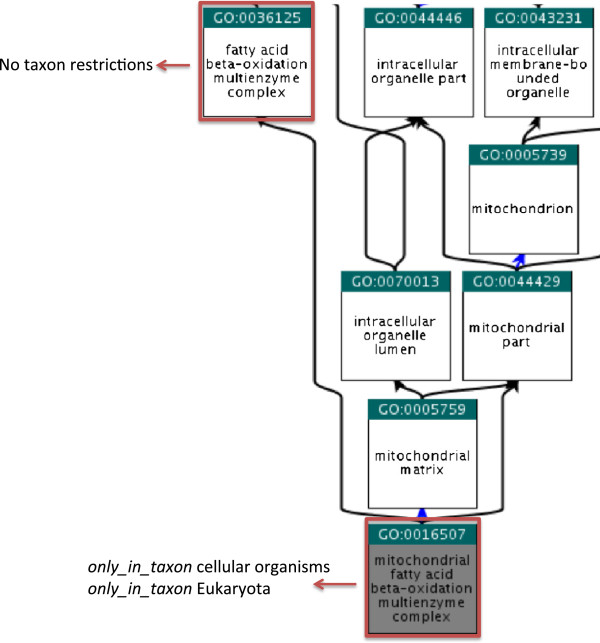
**Inheritance of taxon restrictions.** Less specific, parent terms have fewer taxon restrictions than more specific child terms that are further down the hierarchy. This should be considered when choosing GO terms to use in automatic prediction methods. In the example shown, predicting the term “fatty acid beta-oxidation multienzyme complex” for a set of multispecies proteins may result in more accurate annotation than predicting the term “mitochondrial fatty acid beta-oxidation multienzyme complex”.

Although those annotations that violate a taxon restriction could be removed upon import of the annotations into the UniProt-GOA database, it is always optimal to address the problem at source and ensure that the error is not propagated further. UniProt has been actively working with InterPro, who provide 49% of total GO annotations in the UniProt-GOA database, to assist them in refining the mapping between InterPro identifiers and GO terms, thereby reducing the number of InterPro GO annotations with taxon violations. In many cases, this refinement involved choosing a GO term for the mapping that is further up the hierarchy and is less restrictive with respect to the taxonomic groups it can be used with. This will ensure the predicted annotation is correct over a larger number of gene products. Figure 3 shows an example of how a GO term, in this case 'mitochondrial fatty acid beta-oxidation multienzyme complex’ (GO:0016507), which provides quite specific information, has more taxon restrictions than the less specific term 'fatty acid beta-oxidation multienzyme complex’ (GO:0036125). The InterPro entry IPR012799, which contains matches to Eukaryotic and bacterial proteins, was originally mapped to 'mitochondrial fatty acid beta-oxidation multienzyme complex’ (GO:0016507) causing the bacterial proteins to be mis-annotated. Choosing the parent term 'fatty acid beta-oxidation multienzyme complex’ (GO:0036125) for mapping to GO will result in more accurate annotation for the entire set of protein matches.

Groups providing UniProt with annotations (e.g., InterPro, Ensembl and Ensembl Genomes) are starting to use these taxon restrictions when creating their annotation sets, so providing us with an improved annotation set already from source. The taxon restrictions are also used by the UniProt-developed GO annotation curation interface, Protein2GO [31], to prevent inappropriate manual annotation from being created. Increasing numbers of GO curators from the GOC, as well as some groups external to the GOC, use Protein2GO, thus having quality control mechanisms in place at the time an annotation is created is important to these groups as it significantly reduces annotation errors.

As this will be an ongoing quality assurance process with further annotation providers implementing taxon constraints in their resource, this is expected to result in increasing numbers of annotations being removed from datasets giving a reduced, but more accurate, set of annotation predictions.

### Post-processing of annotations using taxon restrictions

In some cases, however, it is not always possible for the annotation-providing group to refine their mappings between GO and another vocabulary to remove all of the incorrect automatic predictions without an unacceptably large loss of correct annotations. In these cases, UniProt has introduced additional measures to prevent certain gene product-GO term combinations from being created and these are described below.

**Figure 4:**
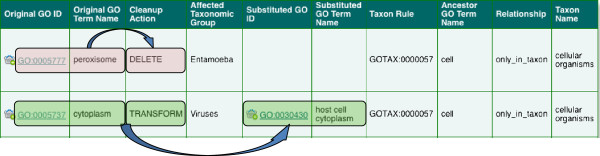
**Post-processing of automatic annotations.** UniProt have rules in place such that if the taxon restrictions are violated in automatic annotations, the annotation can be either deleted (row 1) or edited to use a more appropriate GO term (row 2). In row 1, an *Entamoeba* protein is annotated to “peroxisome”, these organelles are only present in cellular organisms therefore the annotation is deleted. In row 2, a viral protein is annotated to “cytoplasm”, for viruses the correct GO term to use is “host cell cytoplasm” therefore the GO term is substituted and a GO reference describing this editing process is supplied with the annotation.

Post-processing of annotations can ensure taxonomic correctness of annotated GO terms using data supplied by the GO taxon restrictions. An example of when this post-processing can improve the accuracy of annotations is again within the collaboration between UniProt and InterPro. As described in the last section, mappings between InterPro identifiers and GO terms can be refined so that predicted annotations are true for all proteins matched. However, there can be exceptions where the predictions are not applicable to all the proteins matched, for example when a small fraction of family members have lost the active residues and are no longer catalytically active. Removing the mappings between the InterPro identifier and GO term in these cases would result in a large number of valid annotations being deleted, so procedures to handle the small number of inaccurate annotations are invaluable. UniProt has mechanisms in place to automatically handle annotations that fall into this category by utilizing the GO taxon restrictions. Annotations can be either deleted if no suitable alternative GO term can be assigned, or edited to use a more appropriate GO term. Two examples of this are shown in Figure 4. Firstly, an annotation to 'peroxisome’ (GO:0005777) that is predicted for a protein from the Entamoeba taxonomic group is deleted because this group of organisms do not have this specific organelle. The second example is the prediction of 'cytoplasm’ (GO:0005737) for viral proteins when the more accurate term is 'host cell cytoplasm’ (GO:0030430); in this case the GO term is automatically substituted. These updates are reflected in the GO reference that is provided with the annotation.

### Annotation blacklist

The annotation blacklist allows us to specify which protein:GO term combinations should not exist as annotations and it is populated from three sources: 1) curator-review of automatic annotation pipelines, 2) manually curated “caution” comments from UniProt that indicate when a protein does not have the expected function, process or location and 3) NOT annotations provided to UniProtKB entries (annotations that explicitly state a gene product is experimentally shown not to be associated with the annotated Biological Process, Molecular Function or Cellular Component). The blacklist is used by UniProt to prevent these annotations being created not only by automatic annotation, but also by manual annotation through the use of on-the-fly checks in our curation interface, Protein2GO. The annotation blacklist is publicly available as a webservice [32] and we welcome suggestions for further additions.

## Conclusions

We have described here some examples of how the Gene Ontology and its associated annotations can change over time, using examples of how UniProt manages these changes. It is of particular importance to those researchers who make use of GO data for analysis to understand why these changes occur in order to make the appropriate conclusions for their interpretations. Additionally it is important for those who are generating GO annotation to ensure they are using the most up-to-date and appropriate terms. The GOC and the groups who provide GO annotation are continually looking for ways to enhance both the content of the GO and the GO annotations in order to prevent this data from becoming stale, as well as to assist researchers in forming hypotheses based on current and accurate information.

### Endnotes

^a^Search of PubMed using the phrase “Gene Ontology”.

## Abbreviations

GO: Gene Ontology; GOC: Gene Ontology Consortium; MTOC: Microtubule organizing centers; UniProt: Universal Protein Resource; UniProt-GOA: UniProt Gene Ontology Annotation project.

## Competing interests

The authors declare that they have no competing interests.

## Authors’ contributions

TS implemented the annotation post-processing and annotation blacklist procedures used by UniProt-GOA and also developed the UniProt tools, QuickGO and Protein2GO, to incorporate the quality assurance mechanisms described in this paper. All authors contributed to discussions for implementing quality assurance mechanisms within the UniProt-GOA database and its tools. RPH wrote the manuscript. CO’D contributed text for the manuscript. All authors read and approved the final manuscript.

## Authors’ information

RPH is Project Leader of the UniProt-Gene Ontology Annotation project and an Annotation Manager for the GO Consortium since 2012.

TS is Software Engineer of the UniProt-Gene Ontology Annotation project since 2009.

CO’D is Team Leader of UniProt Content since 2009.

MJ-M is Team Leader of UniProt Development since 2009.
